# Book Notices

**Published:** 1866-02

**Authors:** 


### BOOK NOTICES.

*Obscure Diseases of the Brain and Mind.* By FORBES WINSLOW, M. D., D. C. L., etc., etc. Philadelphia: Henry C. Lea. 1866.

Since the publication of Burton's famous Anatomy of Melancholy, a volume more fascinating than this has not appeared. The deserved reputation of its author is alone sufficient to command the attention of the professional public, while the intrinsic importance of its subject cannot fail to attract every student of medicine, who has a relish for anything above the ordinary level of pill-and-powder books. The study of the brain, that wonderful apparatus through which the invisible spirit of man acts upon his corporeal frame, ranks at the head of all material studies. It is the indispensable preliminary to all rational progress in purely intellectual investigation of the laws of mind and the nature of the soul; yet, how often is it neglected by those who profess to spend their lives in the analysis of psychological phenomena. Hence the vagaries of spiritualists, mesmerists and mystics of every degree. Most illustrious is the contrary example of Sir William Hamilton, who, to prepare himself for a discussion of the pretensions of phrenology, examined the brains of not less than three thousand subjects. It is by such researches that the true relations of mind and matter are to be determined, and it is in this way that, while seeking to avoid the Scylla of materialism, we fall not into the Charybdis of ultra idealism.

If then the brain is so important an organ, the observation of its disorders must be of paramount importance. To guide the student of cerebral and psychological pathology, is the object of Dr. Winslow. He endeavors to point out the reciprocity of influence which exists between mind and matter, and to suggest the measures which, by warding off disease, or by removing its effects from the material instrument, may preserve unfettered that spiritual agent which is Man himself. "We should never lose sight of the fact that no irritation or inflammatory action can exist for any length of time, in the more important tissues, or ganglia of the brain, without seriously perilling the reason and endangering life. . . . A vast and frightful amount of chronic and incurable insanity exists at this moment in our county and private asylums, which can be clearly traced to the criminal neglect of the disease in the first or incipient stage." *p.* 28. "Men have been hanged for crimes which the physician might have prevented. Many a suicide would be prevented, and murderous and criminal impulse destroyed, if an active cathartic were exhibited, or the cerebral circulation relieved and rendered less active by means of local depletion. Damien declared to the last that, had the vessels of his brain been unloaded by bleeding, as he earnestly requested, he never would have attempted the life of Louis XV." *p.* 121.

Every medical man can recall the details of many a case where the happiness of the family has been destroyed, the decorum of the church disturbed, and the peace of society outraged, by individuals who have been anathematized by the good, and visited with all the penalties of the law for offences which were innocently committed by the victims of obscure disease of the brain, which required the careful attention of the physician, instead of the harrowing censures of courts, civil or ecclesiastical. Many such histories are related by Dr. Winslow, who, however, wisely insists that the plea of insanity should never be allowed to enlarge the defendant who stands convicted of the crime. The criminal whose innocence is established by the proof of insanity, should be ordered to the asylum—he is not fit to remain at liberty.

Our limits will not admit a more extended notice of this admirable work. It cannot fail to interest even the unprofessional reader, for though its subject allies it to profoundest treatises on mental philosophy, the narratives, which illustrate the theme and crowd the pages of the volume, are as absorbing as the wildest inventions of fiction.

---

*The Medical Record.*—The first number of this new medical periodical has reached us. It is a semi-monthly journal of twenty-four pages, published by the well-known firm of Wm. Wood & Co., New York. Dr. Geo. F. Shrady, formerly associate editor of the *American Medical Times*, is the editor. The journal in general appearance and arrangement, resembles the *London Medical Times and Gazette*, and promises to constitute one of the most valuable of our exchanges.

---

New Form of Stem Pessary.—This instrument, of which our readers will find an illustration in the advertising sheet of the Journal, is recommended by many eminent obstetricians, and is worthy of a trial by the profession. It is manufactured of vulcanized rubber by Drs. Boyd and Ferree at Indianapolis, Indiana.

